# Supplementary figures and images for: MiCloud: A unified web platform for comprehensive microbiome data analysis
Source: PLoS One. 2022 Aug 1;17(8):e0272354. doi: 10.1371/journal.pone.0272354 (PMC9342768; doi:10.1371/journal.pone.0272354)

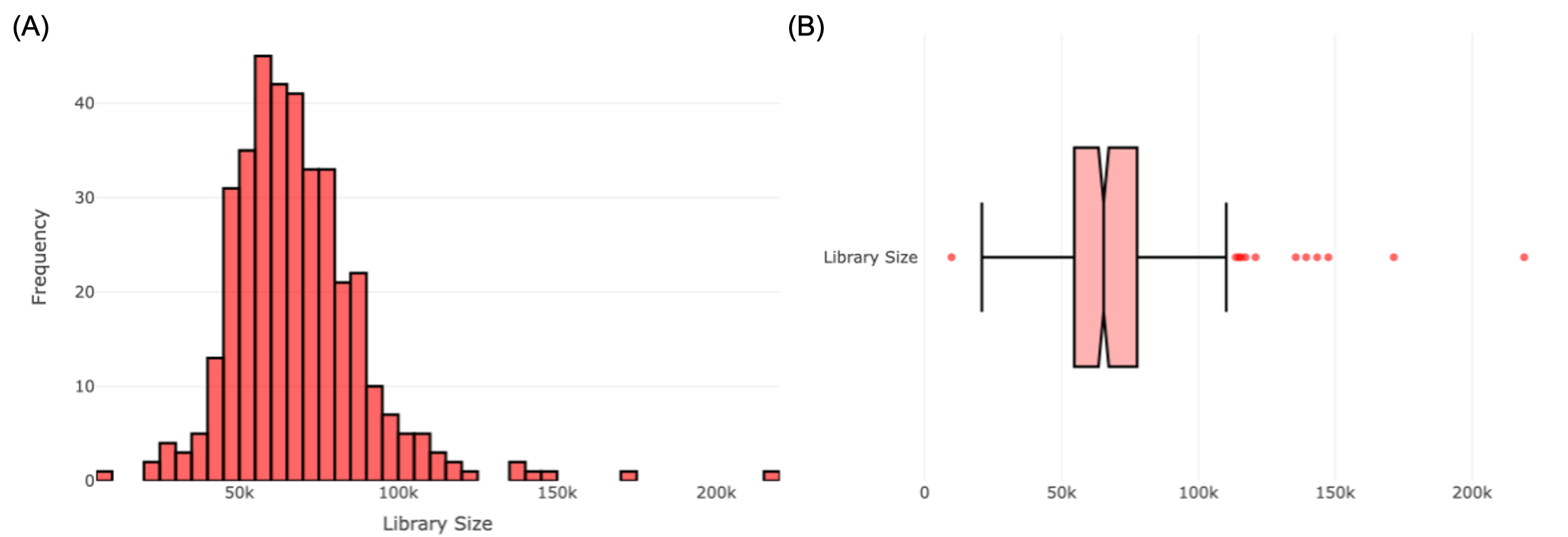

Supplement: S1 Fig — The histogram (A) and box plot (B) for library sizes across subjects after quality controls. (TIF) [file pone.0272354.s001.tif]

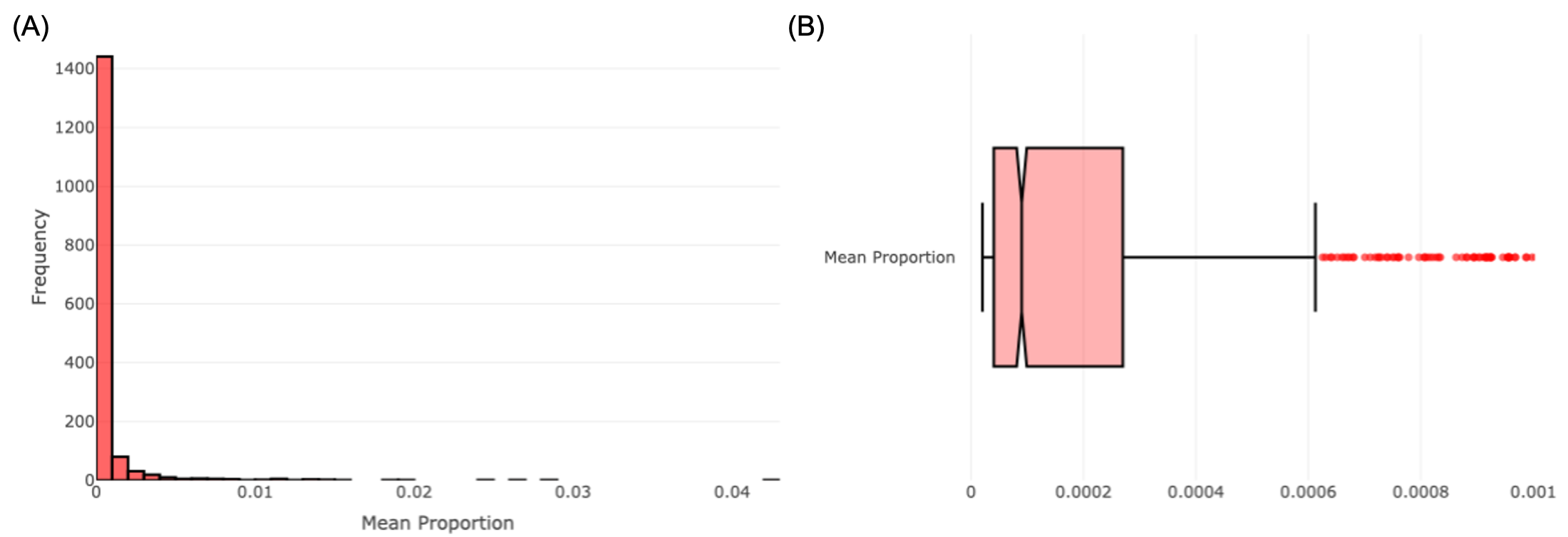

Supplement: S2 Fig — The histogram (A) and box plot (B) for mean proportions across OTUs after quality controls. (TIF) [file pone.0272354.s002.tif]
